# Supplementary material for: Mapping DNA damage‐dependent genetic interactions in yeast via party mating and barcode fusion genetics
Source: Mol Syst Biol. 2018 May 28;14(5):e7985. doi: 10.15252/msb.20177985 (PMC5974512; doi:10.15252/msb.20177985)
Supplement: Supplementary file 1 — Expanded View Figures PDF [file MSB-14-e7985-s001.pdf]

Expanded View Figures

Figure EV1. Donor toolkit construction.

A Two fragments were built to generate proDonor plasmids. The first, preD1, contained *loxP*/*lox2272* sites flanking two 20-bp unique barcodes and a hygromycin resistance marker. In this study, only the upstream barcode was used for further steps, and for simplification, the downstream barcode was omitted from Fig 1.

B The second, preD2, contained the Cre recombinase driven by the doxycycline-inducible tetO-CMV, and a *URA3* marker.

C The two fragments were assembled *in vivo* in yeast to generate pDonors.

D pDonors were arrayed and Sanger-sequenced to confirm the integrity of the preD1 fragment. ProDonors with confirmed preD1 fragments were mated with YKO strains to generate strains carrying both a uniquely barcoded pDonor and a gene deletion of interest. Then, they were sporulated and the haploid *MAT*alpha progeny were selected using the mating type maker indicated in panel (C). Details on selective media are shown in Fig EV3.

© 2018 The Authors

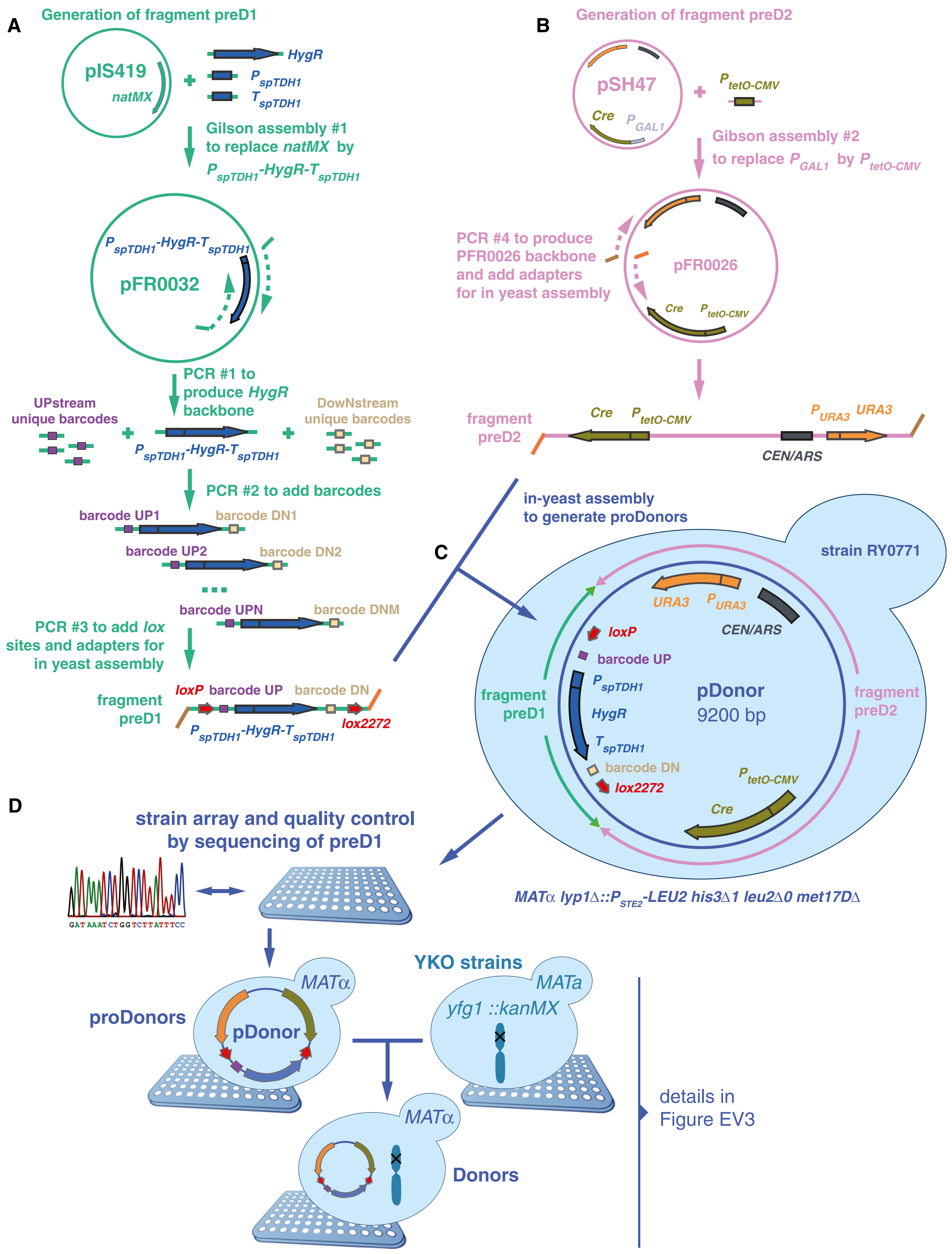
A
Generation of fragment preD1
pIS419
natMX
HygR
PspTDH1
TspTDH1
Gilson assembly #1
to replace natMX by
PspTDH1-HygR-TspTDH1
PspTDH1-HygR-TspTDH1
pFR0032
PCR #1 to
produce HygR
backbone
UPstream
unique barcodes
DowNstream
unique barcodes
PspTDH1-HygR-TspTDH1
PCR #2 to add barcodes
barcode UP1
barcode DN1
barcode UP2
barcode DN2
barcode UPN
barcode DNM
PCR #3 to add lox
sites and adapters for
in yeast assembly
fragment
preD1
loxP
barcode UP
barcode DN
lox2272
PspTDH1-HygR-TspTDH1
B
Generation of fragment preD2
pSH47
Cre
PGAL1
PtetO-CMV
Gibson assembly #2
to replace PGAL1 by PtetO-CMV
PCR #4 to produce
PFR0026 backbone
and add adapters
for in yeast assembly
pFR0026
Cre
PtetO-CMV
fragment
preD2
Cre
PtetO-CMV
PURA3
URA3
CEN/ARS
in-yeast assembly
to generate proDonors
C
strain RY0771
URA3
PURA3
CEN/ARS
loxP
barcode UP
PspTDH1
HygR
TspTDH1
barcode DN
lox2272
pDonor
9200 bp
PtetO-CMV
Cre
fragment
preD1
fragment
preD2
MATα lyp1Δ::PSTE2-LEU2 his3Δ1 leu2Δ0 met17DΔ
D
strain array and quality control
by sequencing of preD1
GATAAATCTGGTCTTATTTCC
proDonors
MATα
pDonor
YKO strains
MATa
yfg1 ::kanMX
MATα
Donors
details in
Figure EV3

Figure EV1.

Figure EV2. Recipient toolkit construction.

A Two constructs were built to generate recipients. The first fragment, preR1, contained *loxP/lox2272* sites flanking a klURA3 marker and two 20-bp unique barcodes flanking these loci. In this study, only the upstream barcode was used for further steps, and for simplification, the downstream barcode was omitted from Fig 1.

B The second construct, preR2, contained the *can1Δ::*P_{STE2}*-spHis5-*T_{STE2} mating type marker.

C The two fragments were assembled *in vivo* using a derivative of the *delitto perfetto* construct.

D Resulting proRecipients were arrayed and Sanger-sequenced to confirm integrity of preR1 loci. ProRecipients with confirmed preR1 loci were mated with SGA query strains to generate strains carrying both a uniquely barcoded recipient construct and a gene deletion of interest. Then, they were sporulated and the haploid *MAT***a** progeny were selected using the mating type maker indicated in panel (C). Details on selective media are shown in Fig EV3.

© 2018 The Authors

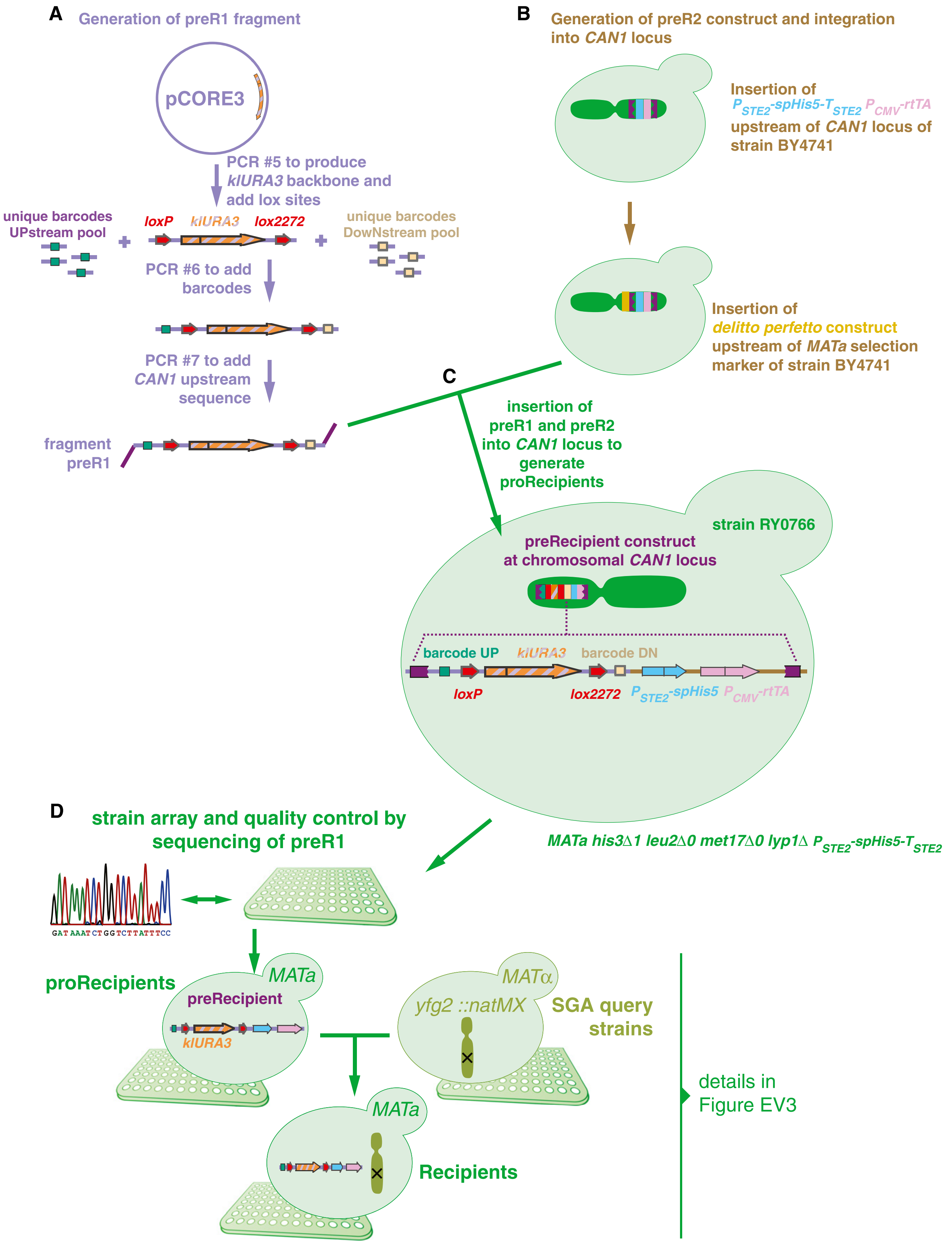
A
Generation of preR1 fragment
pCORE3
PCR #5 to produce
klURA3 backbone and
add lox sites
unique barcodes
UPstream pool
loxP
klURA3
lox2272
unique barcodes
DowNstream pool
PCR #6 to add
barcodes
PCR #7 to add
CAN1 upstream
sequence
fragment
preR1
B
Generation of preR2 construct and integration
into CAN1 locus
Insertion of
PSTE2-spHis5-TSTE2 PCMV-rtTA
upstream of CAN1 locus of
strain BY4741
Insertion of
delitto perfetto construct
upstream of MATa selection
marker of strain BY4741
C
insertion of
preR1 and preR2
into CAN1 locus to
generate
proRecipients
strain RY0766
preRecipient construct
at chromosomal CAN1 locus
barcode UP
klURA3
barcode DN
loxP
lox2272
PSTE2-spHis5 PCMV-rtTA
MATa his3Δ1 leu2Δ0 met17Δ0 lyp1Δ PSTE2-spHis5-TSTE2
D
strain array and quality control by
sequencing of preR1
GATAAATCTGGTCTTATTTCC
proRecipients
MATa
preRecipient
klURA3
MATα
yfg2 ::natMX
SGA query
strains
details in
Figure EV3
MATa
Recipients

Figure EV2.

Figure EV3. Media details to generate BFG-GI strains and pools.

Donors, recipients, and double mutants used in BFG-GI were generated as shown in Figs 1, EV1 and EV2. This figure shows media details, optimal inoculum cell densities, and incubation times for pool-based cultures. All incubations were at 30°C for 24 h, except for mating (12 h at 23°C) and sporulation (12 days at 21°C). Sporulation was conducted in flasks with liquid media shaking at 200 rpm. We used the following reagent concentrations: G418 = 200 μg/ml; clonNat = 100 μg/ml; canavanine = 100 μg/ml; thialysine = 100 μg/ml; hygromycin = 200 μg/ml; and 5-FOA = 1 mg/ml. Amino acid concentrations were as described in Tong and Boone (2005).

© 2018 The Authors

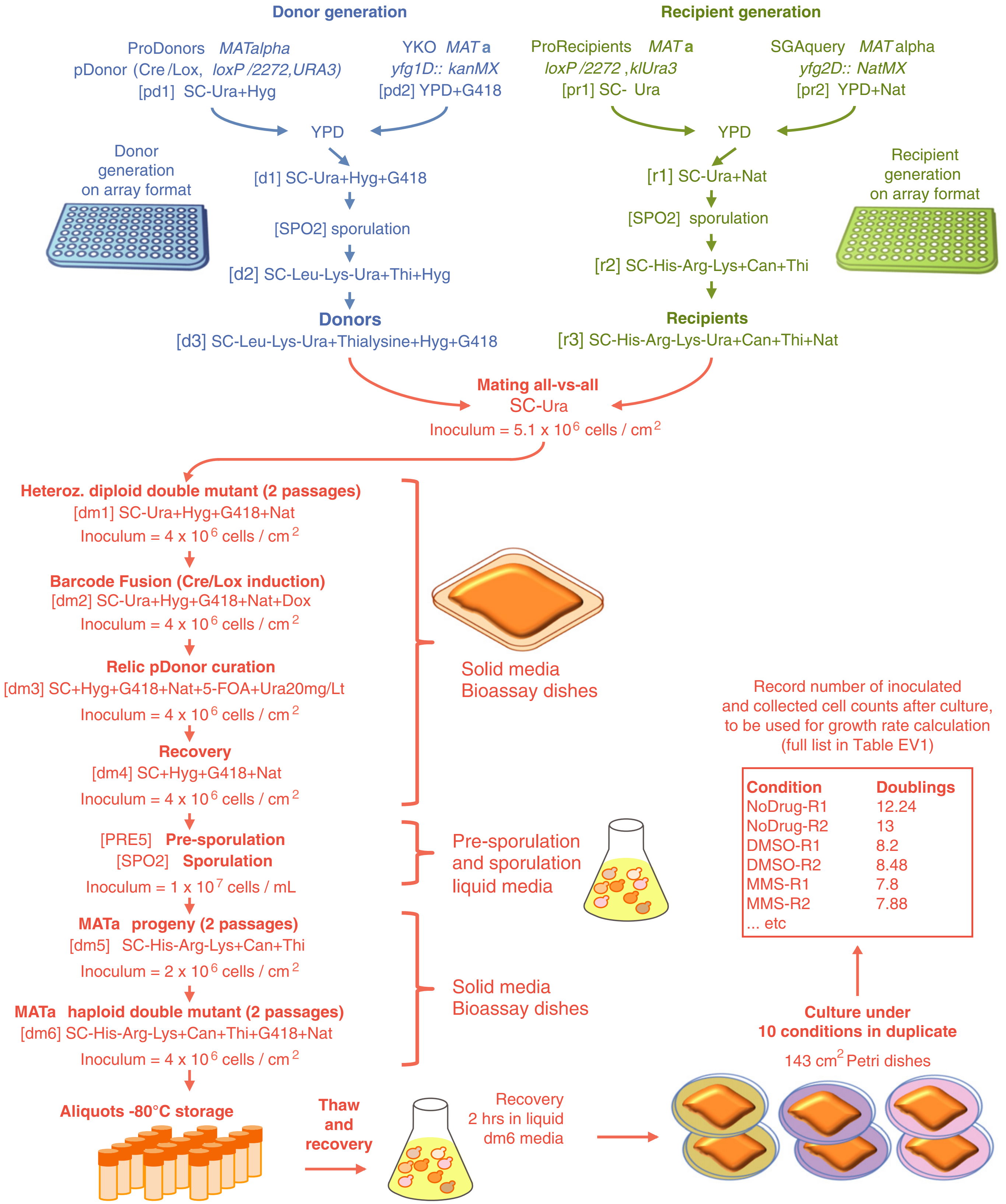
Donor generation
ProDonors MATalpha
pDonor (Cre/Lox, loxP/2272,URA3)
[pd1] SC-Ura+Hyg
YKO MATa
yfg1D:: kanMX
[pd2] YPD+G418
YPD
Donor generation on array format
[d1] SC-Ura+Hyg+G418
[SPO2] sporulation
[d2] SC-Leu-Lys-Ura+Thi+Hyg
Donors
[d3] SC-Leu-Lys-Ura+Thialysine+Hyg+G418
Recipient generation
ProRecipients MATa
loxP/2272,klUra3
[pr1] SC- Ura
SGAquery MATalpha
yfg2D:: NatMX
[pr2] YPD+Nat
YPD
[r1] SC-Ura+Nat
Recipient generation on array format
[SPO2] sporulation
[r2] SC-His-Arg-Lys+Can+Thi
Recipients
[r3] SC-His-Arg-Lys-Ura+Can+Thi+Nat
Mating all-vs-all
SC-Ura
Inoculum = 5.1 x 10^6 cells / cm^2
Heteroz. diploid double mutant (2 passages)
[dm1] SC-Ura+Hyg+G418+Nat
Inoculum = 4 x 10^6 cells / cm^2
Barcode Fusion (Cre/Lox induction)
[dm2] SC-Ura+Hyg+G418+Nat+Dox
Inoculum = 4 x 10^6 cells / cm^2
Relic pDonor curation
[dm3] SC+Hyg+G418+Nat+5-FOA+Ura20mg/Lt
Inoculum = 4 x 10^6 cells / cm^2
Recovery
[dm4] SC+Hyg+G418+Nat
Inoculum = 4 x 10^6 cells / cm^2
Solid media Bioassay dishes
[PRE5] Pre-sporulation
[SPO2] Sporulation
Inoculum = 1 x 10^7 cells / mL
Pre-sporulation and sporulation liquid media
MATa progeny (2 passages)
[dm5] SC-His-Arg-Lys+Can+Thi
Inoculum = 2 x 10^6 cells / cm^2
MATa haploid double mutant (2 passages)
[dm6] SC-His-Arg-Lys+Can+Thi+G418+Nat
Inoculum = 4 x 10^6 cells / cm^2
Solid media Bioassay dishes
Record number of inoculated and collected cell counts after culture, to be used for growth rate calculation (full list in Table EV1)
Condition Doublings
NoDrug-R1 12.24
NoDrug-R2 13
DMSO-R1 8.2
DMSO-R2 8.48
MMS-R1 7.8
MMS-R2 7.88
... etc
Culture under 10 conditions in duplicate
143 cm^2 Petri dishes
Aliquots -80°C storage
Thaw and recovery
Recovery 2 hrs in liquid dm6 media

Figure EV3.

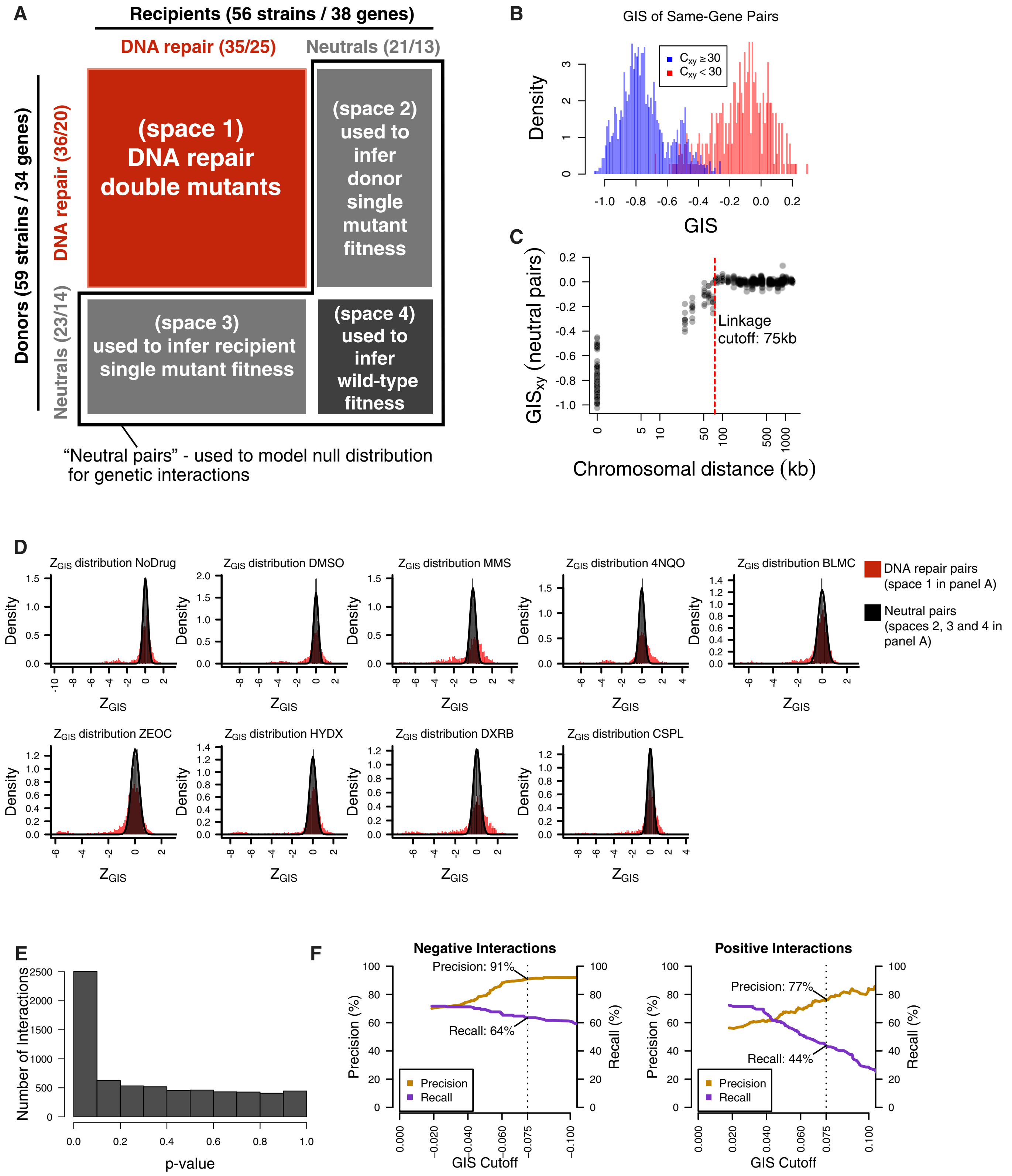

Figure EV4.

 © 2018 The Authors

Figure EV4. Calling genetic interactions.

A Two collections of 59 donor strains (containing 34 unique knockouts) and 56 recipient strains (containing 38 unique gene knockouts) were crossed against each other in an all-by-all pooled format. Each strain contains a knockout at either a DNA repair gene or neutral locus. Double-knockout strains were divided into four spaces based on the types of genes knocked out. Numbers in parentheses represent the number of strains and unique gene knockouts, respectively.

B Distribution of *GIS* amongst strain pairs containing the same gene, split by those which were well-measured from the heterozygous diploid stage ($C_{xy} \geq 30$) and not well-measured from the same stage ($C_{xy} < 30$). Non-well-measured strains (72 out of 3,305) were excluded from analysis, and *GIS* was re-calculated after their exclusion.

C Distribution of *GIS* in strains representing linked neutral pairs. Using the *GIS* profiles, an empirical cutoff of 75 kbp (red dashed line) was chosen to classify strains with knockout pairs on the same chromosome as either linked or unlinked. *GIS* was then re-calculated based on this linkage criterion.

D Distribution of Z_{GIS} calculated for DNA repair pairs (space 1 in panel A, red) and pairs involving well-measured and unlinked neutral genes (spaces 2, 3, and 4 in panel A, black). Z_{GIS} for pairs involving neutral genes were used to calculate a *P*-value.

E Distribution of *P*-values calculated by the null distribution in (D). *P*-values were combined for multiple barcode replicates of each gene–gene pair and converted to FDR scores (see Materials and Methods). Barcode-level *P*-values are available in Table EV3, and gene-level FDR scores are available in Table EV4.

F Benchmarks of BFG-GI with data from St Onge *et al* (2007) for strains containing a significant genetic interaction (FDR < 0.01). Each graph shows precision and recall using the benchmark of St Onge *et al* (2007) as a function of an additional *GIS* effect-size cutoff (left = negative interaction performance; right = positive interaction performance). Overlay text indicates performance at |*GIS*| = 0.075 (dashed lines), which was chosen as the effect-size threshold.

© 2018 The Authors

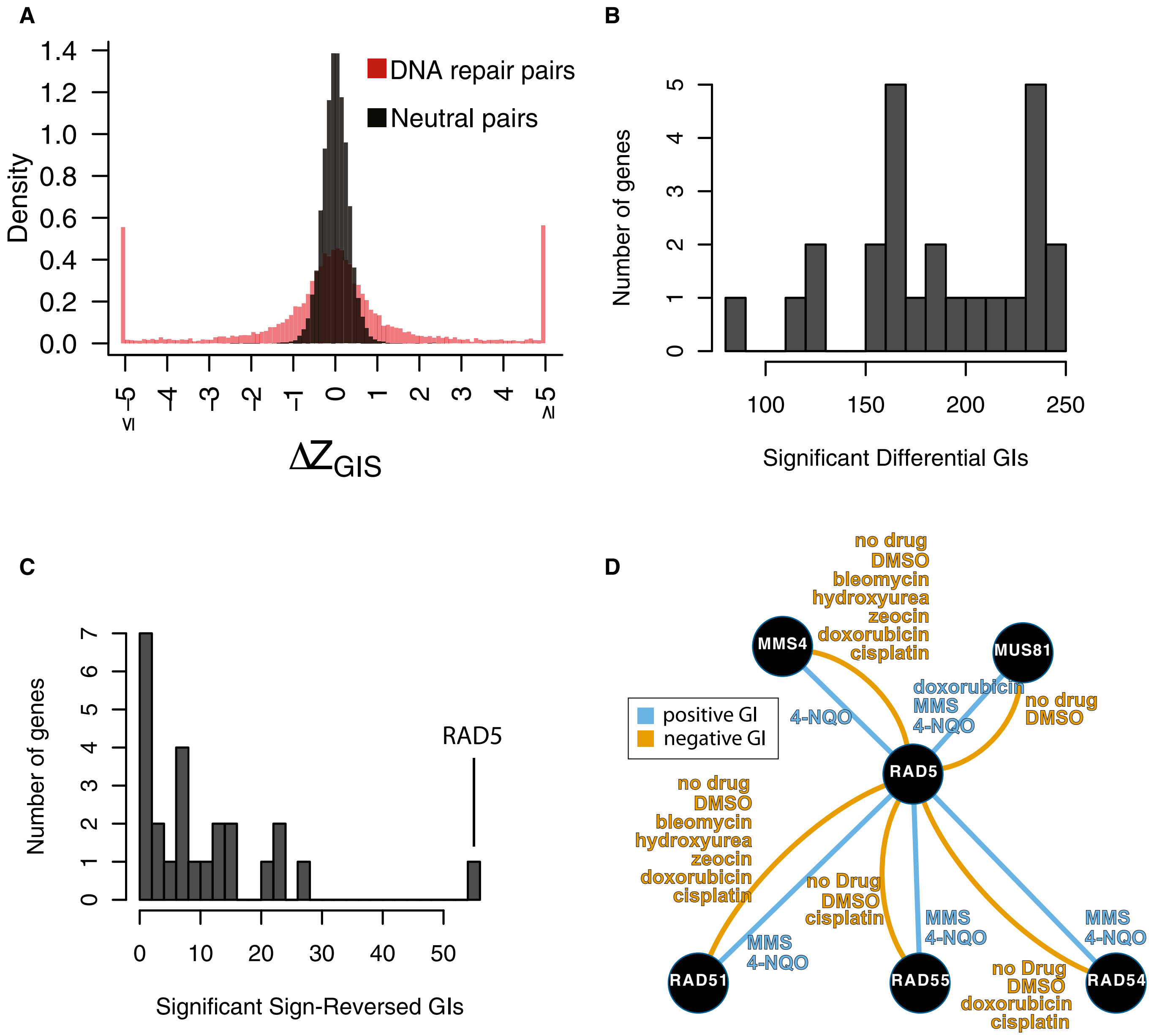

Figure EV5. Calling differential genetic interactions.

A Distribution of ΔZ_{GIS} for neutral pairs compared to DNA repair pairs. The distribution amongst neutral pairs was used to calculate a *P*-value for ΔZ_{GIS} amongst DNA repair pairs, which was then converted to an FDR for each differential interaction (see Materials and Methods; Table EV6). An additional effect-size cutoff of $|\Delta GIS| > 0.1$ was added to call differential genetic interactions in Fig 3 and Table EV5.

B Distribution of significant differential genetic interaction calls per gene.

C Distribution of significant differential genetic interaction calls involving a reversal of direction (i.e., from positive to negative or vice versa) by gene. *RAD5* is involved in 47 differential genetic interactions with a reversal of direction.

D Summary of significant genetic interactions of *RAD5* with *MUS81*, *MMS4*, *RAD51*, *RAD54*, or *RAD55* in different conditions. Edges represent genetic interaction type and are labeled by conditions in which significant genetic interactions were found for the corresponding pair and direction.

 © 2018 The Authors
