# Supplementary material for: Mapping DNA damage‐dependent genetic interactions in yeast via party mating and barcode fusion genetics
Source: Mol Syst Biol. 2018 May 28;14(5):e7985. doi: 10.15252/msb.20177985 (PMC5974512; doi:10.15252/msb.20177985)
Supplement: Supplementary file 8 — Code EV1 [file MSB-14-e7985-s008.zip › Computer_code_EV1_description.rtf]

Computer Code EV1. Contains the computer code used to generate Tables EV3 to EV6 from raw data (Table EV2). Also contains the output of various panels used in Figs 2, 3, EV4, EV5. Any modifications post-publication will have been documented at: https://github.com/a3cel2/BFG_GI_stats.
