# Supplementary figures and images for: Mapping DNA damage‐dependent genetic interactions in yeast via party mating and barcode fusion genetics
Source: Mol Syst Biol. 2018 May 28;14(5):e7985. doi: 10.15252/msb.20177985 (PMC5974512; doi:10.15252/msb.20177985)

NoDrug

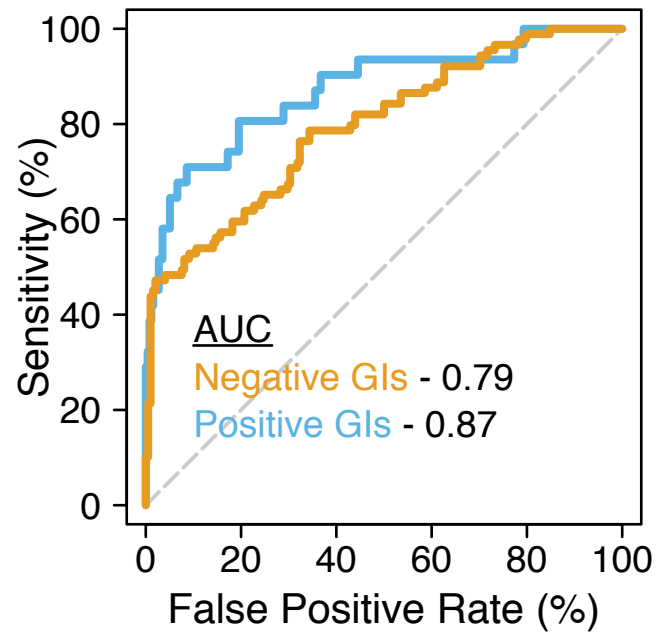

MMS

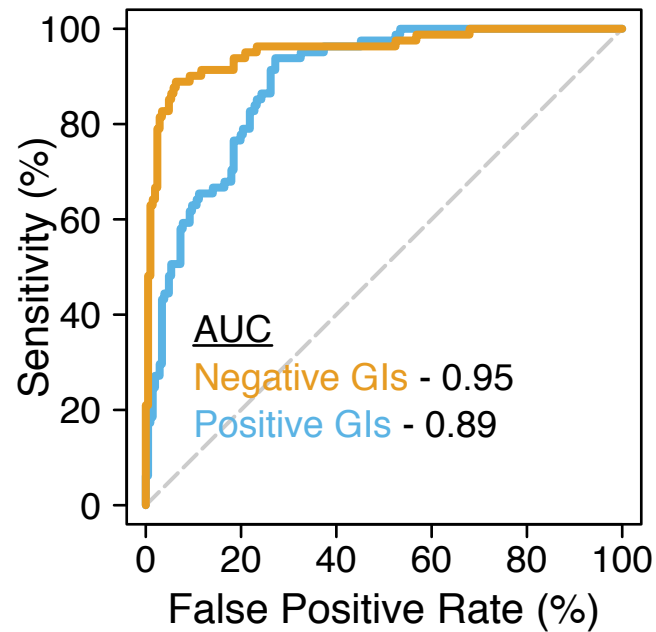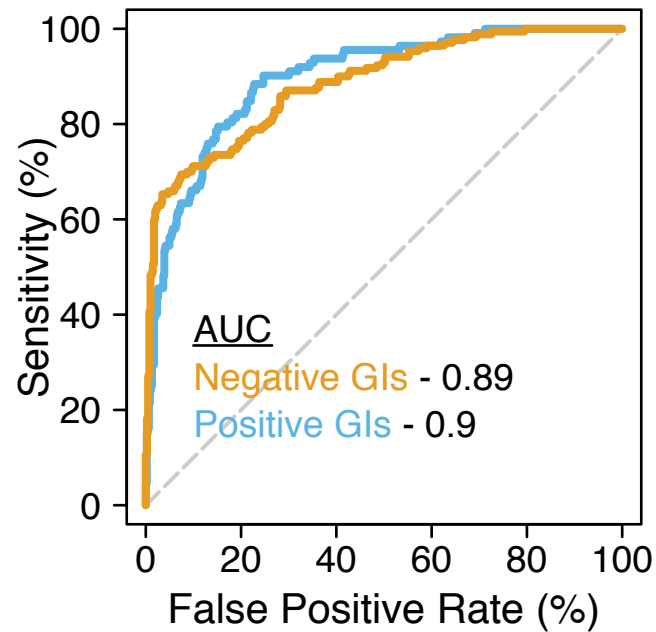

Supplement: Supplementary file 8 — Code EV1 [file MSB-14-e7985-s008.zip › BFG_GI_stats-master/results/auc_vs_st_onge_new.pdf]

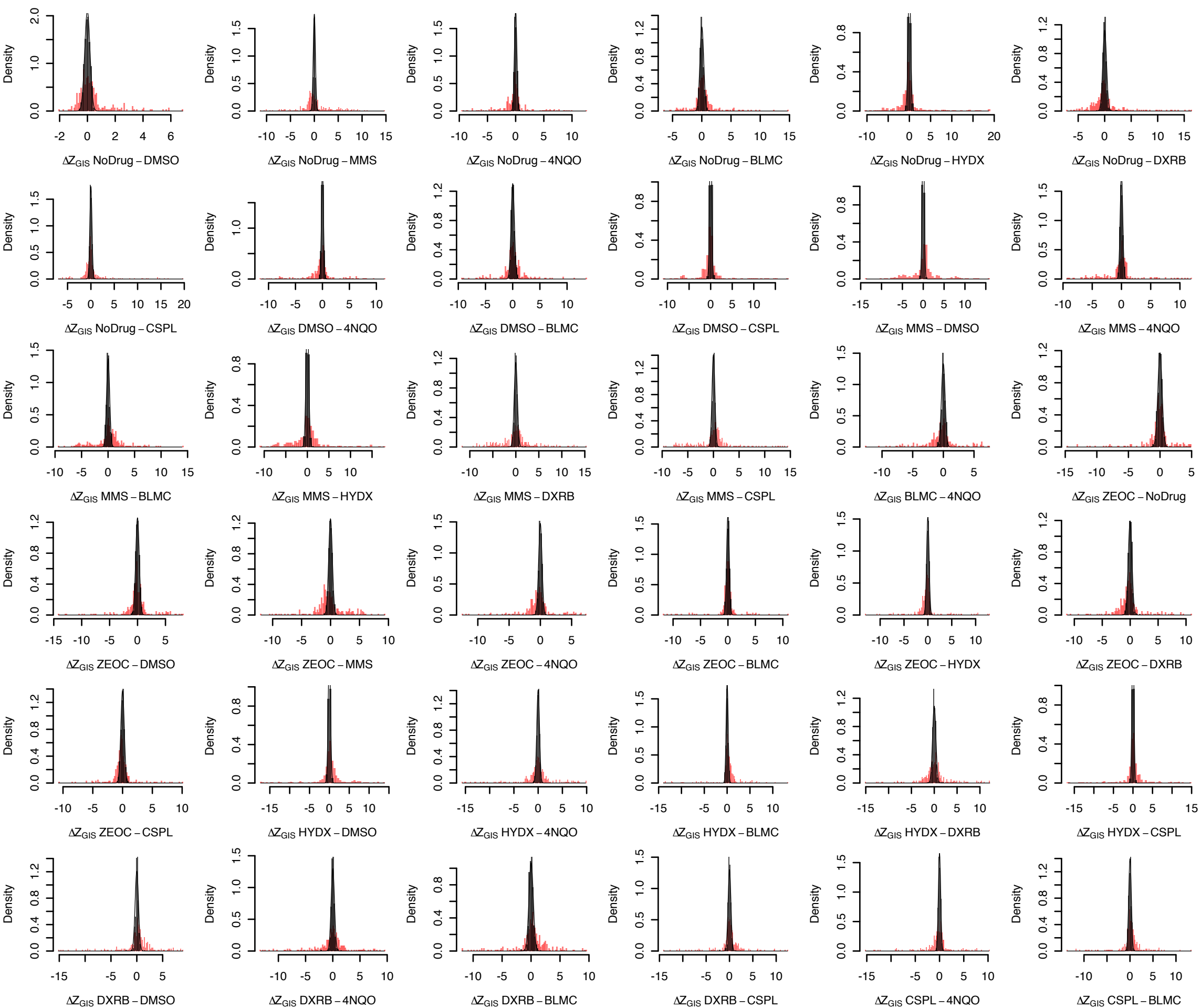

Supplement: Supplementary file 8 — Code EV1 [file MSB-14-e7985-s008.zip › BFG_GI_stats-master/results/delta_z_distribution.pdf]

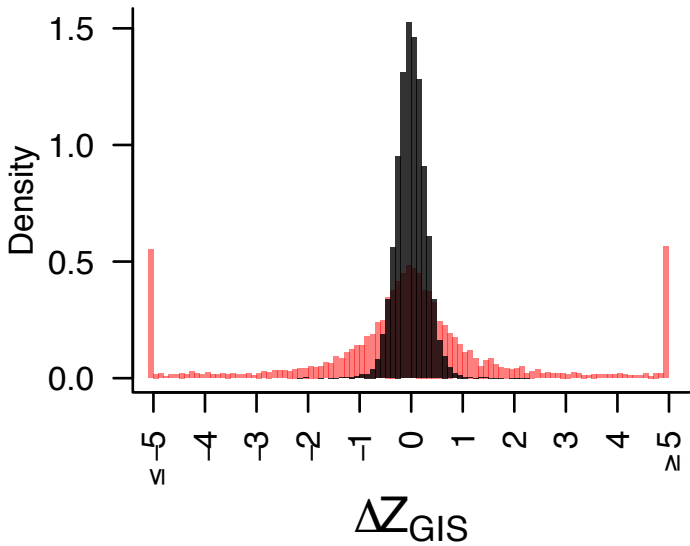

Supplement: Supplementary file 8 — Code EV1 [file MSB-14-e7985-s008.zip › BFG_GI_stats-master/results/delta_z_distribution_overall.pdf]

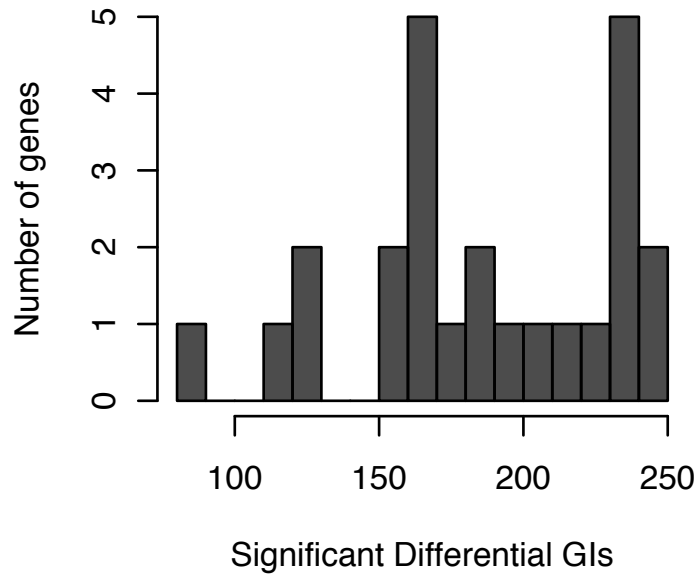

Supplement: Supplementary file 8 — Code EV1 [file MSB-14-e7985-s008.zip › BFG_GI_stats-master/results/differential_inters_by_gene.pdf]

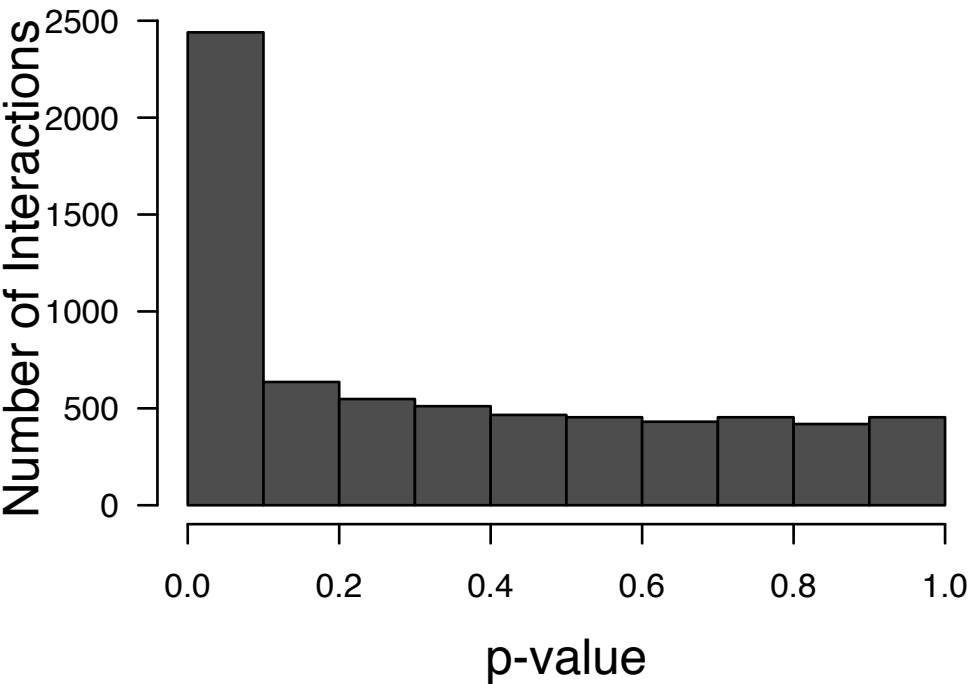

Supplement: Supplementary file 8 — Code EV1 [file MSB-14-e7985-s008.zip › BFG_GI_stats-master/results/gene_averaged_p_value_histogram.pdf]

Prob. density

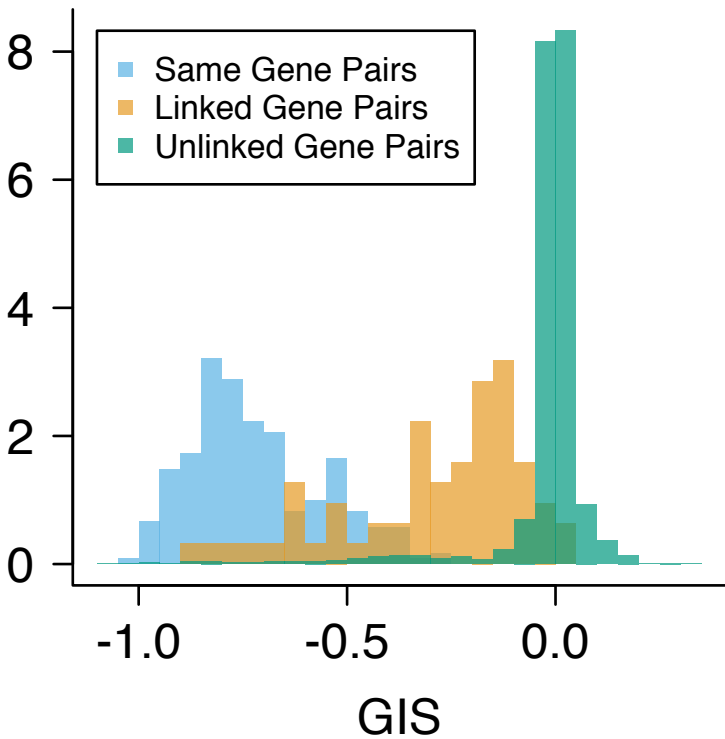

Supplement: Supplementary file 8 — Code EV1 [file MSB-14-e7985-s008.zip › BFG_GI_stats-master/results/GIS_by_linkage.pdf]

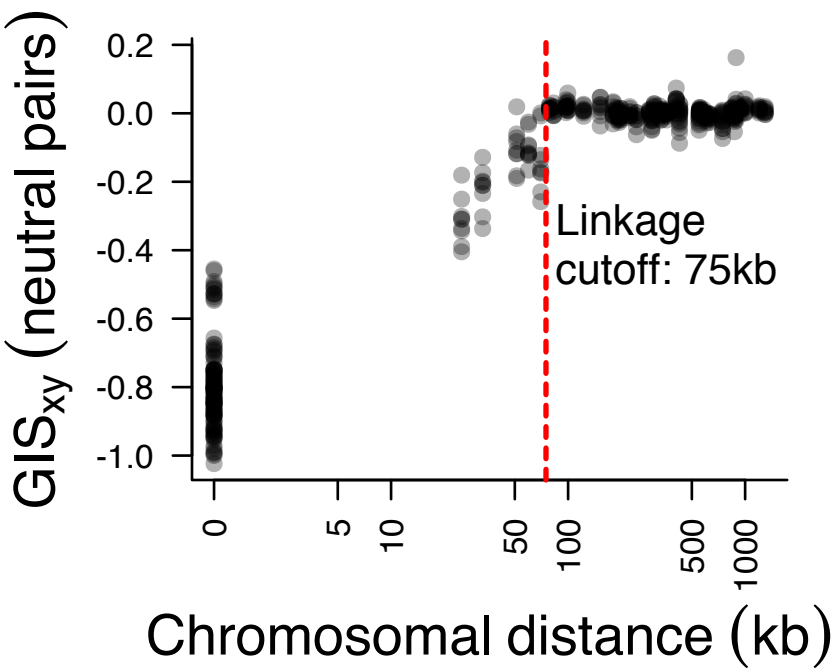

Supplement: Supplementary file 8 — Code EV1 [file MSB-14-e7985-s008.zip › BFG_GI_stats-master/results/GIS_neutrals_vs_distance.pdf]

# GIS of Same-Gene Pairs

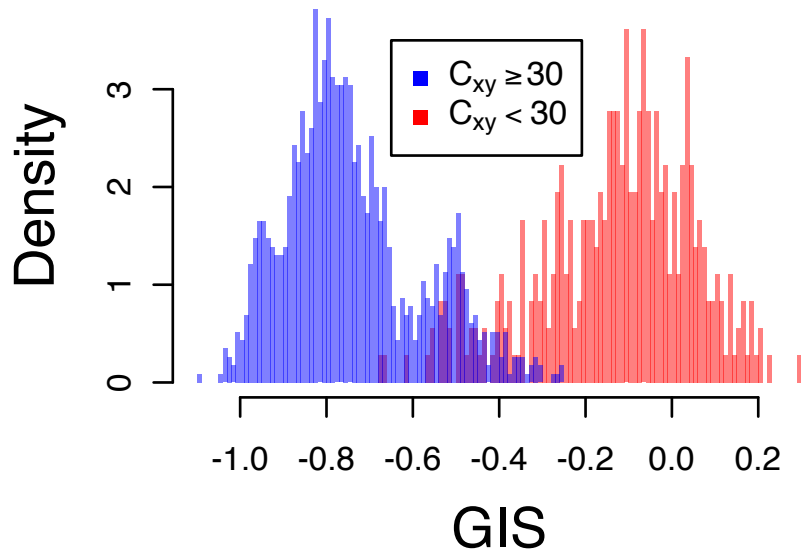

Supplement: Supplementary file 8 — Code EV1 [file MSB-14-e7985-s008.zip › BFG_GI_stats-master/results/gis_well_measured_vs_non.pdf]

## Negative Interactions

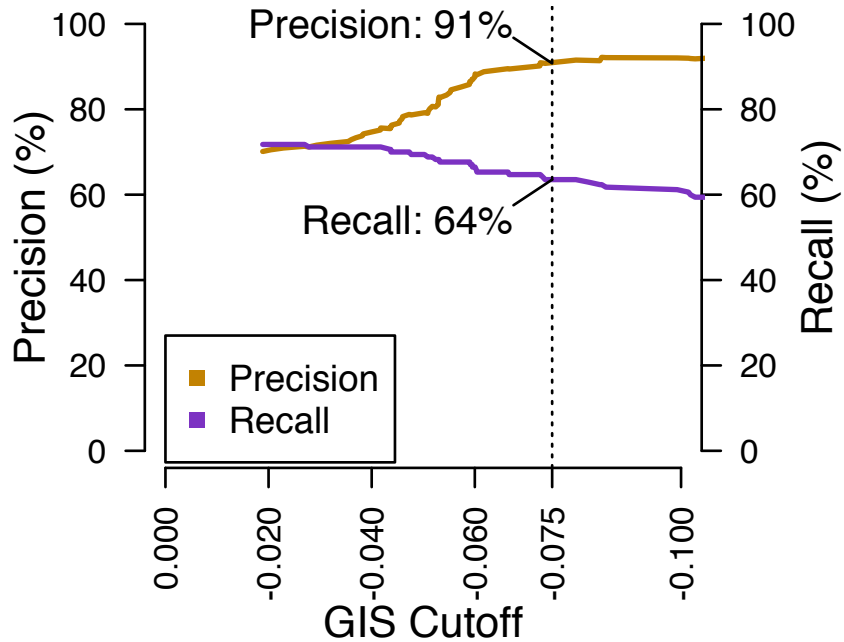

## Positive Interactions

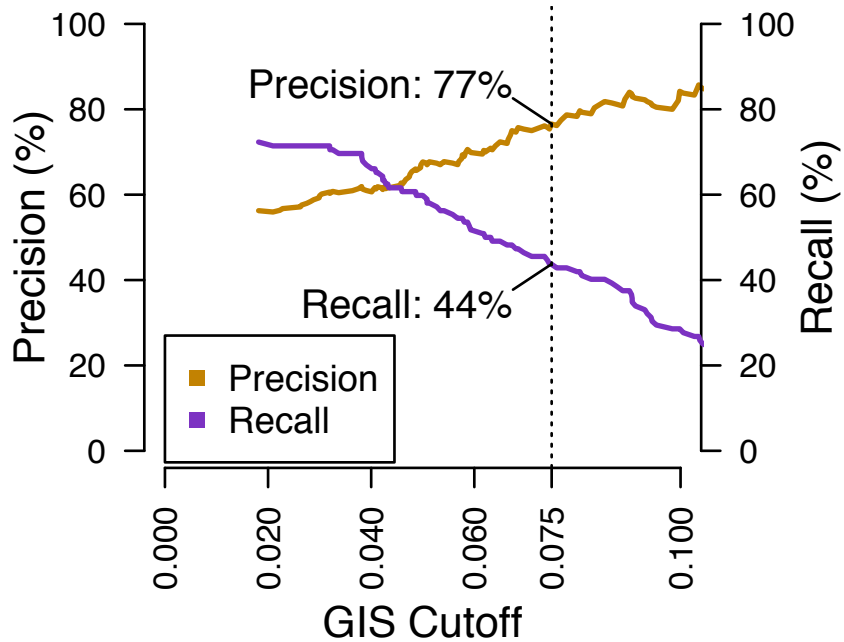

Supplement: Supplementary file 8 — Code EV1 [file MSB-14-e7985-s008.zip › BFG_GI_stats-master/results/prec_rec_vs_st_onge.pdf]

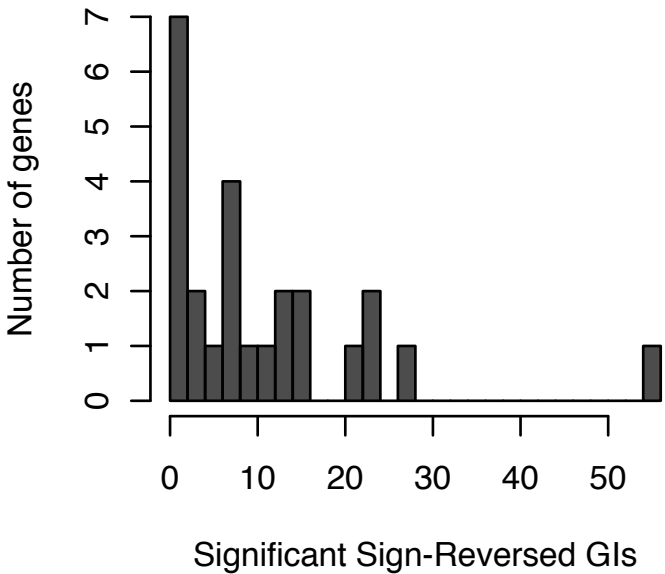

Supplement: Supplementary file 8 — Code EV1 [file MSB-14-e7985-s008.zip › BFG_GI_stats-master/results/sign_reversals_by_gene.pdf]

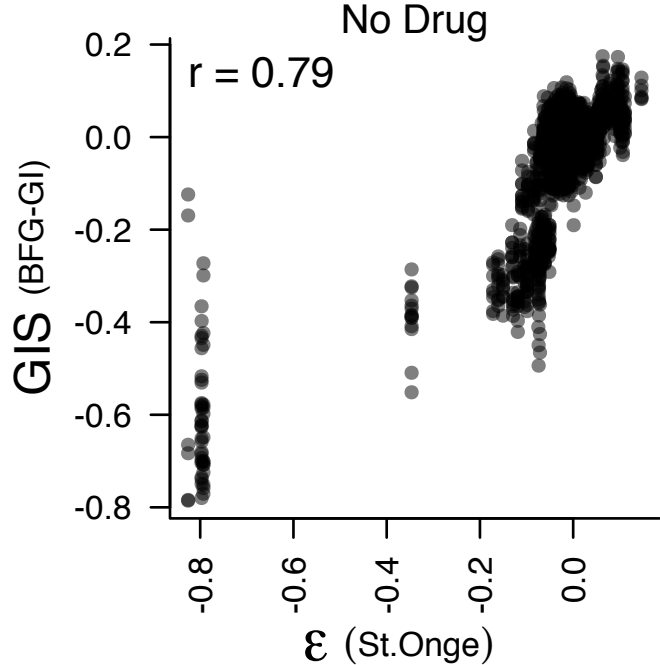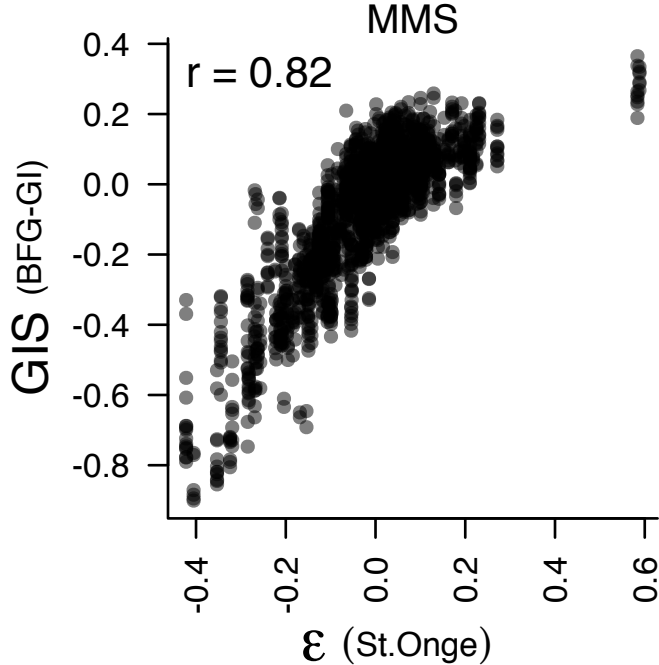

Supplement: Supplementary file 8 — Code EV1 [file MSB-14-e7985-s008.zip › BFG_GI_stats-master/results/st_onge_scatterplot_barcodewise.pdf]

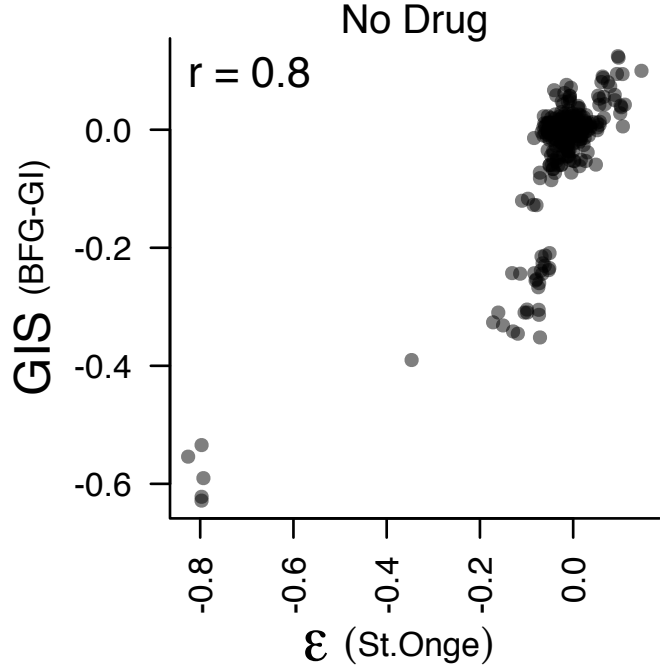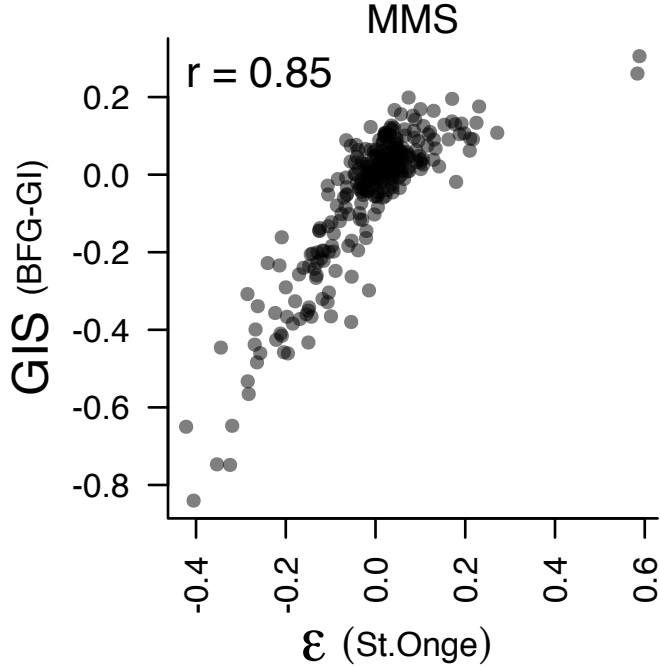

Supplement: Supplementary file 8 — Code EV1 [file MSB-14-e7985-s008.zip › BFG_GI_stats-master/results/st_onge_scatterplot_genewise.pdf]

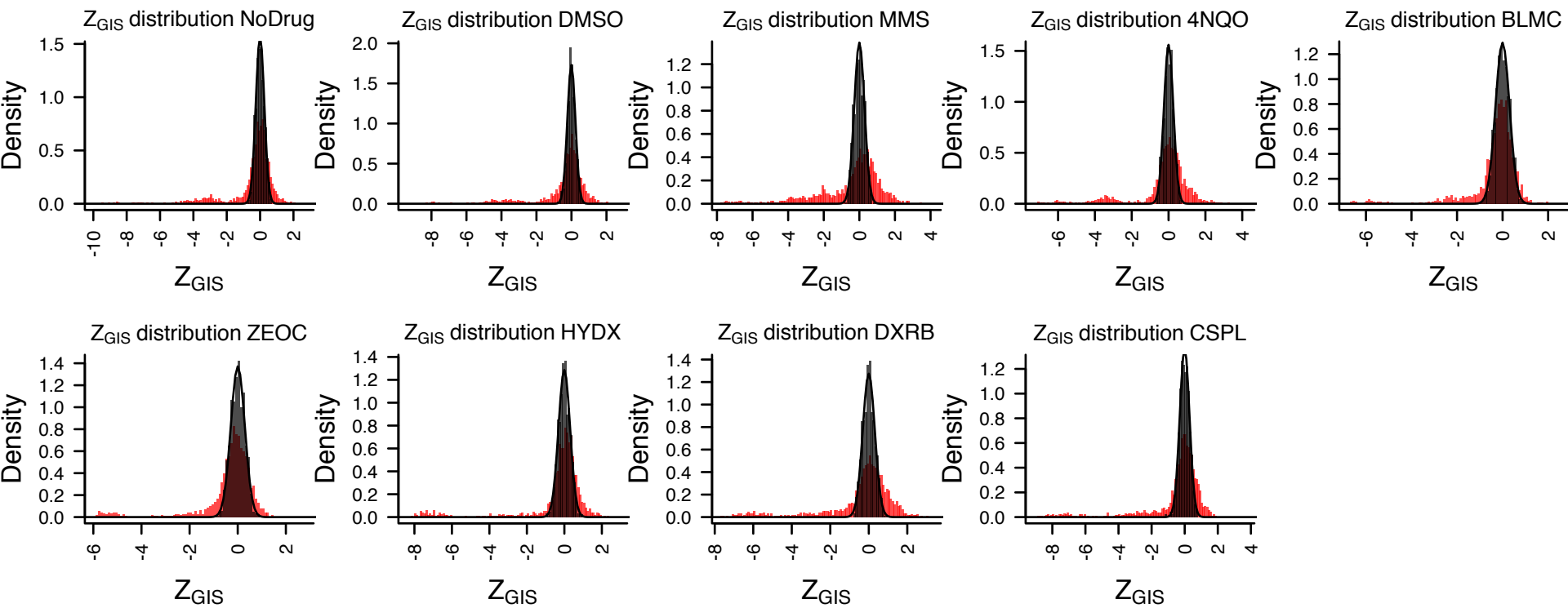

Supplement: Supplementary file 8 — Code EV1 [file MSB-14-e7985-s008.zip › BFG_GI_stats-master/results/Z_distribution.pdf]

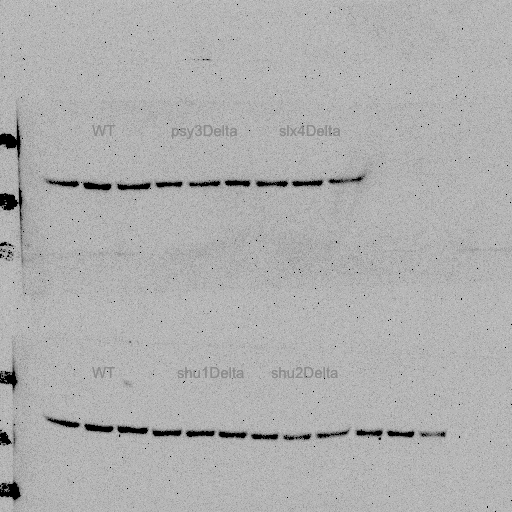

Supplement: Supplementary file 10 — Source Data for Figure 4 [file MSB-14-e7985-s009.zip › Fig_4F_Part1_PGK1.tif]

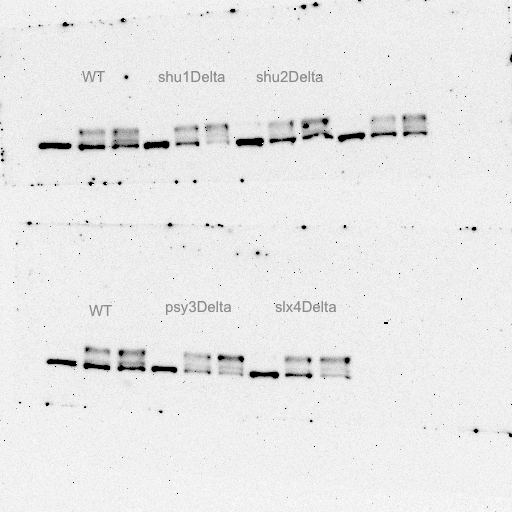

Supplement: Supplementary file 10 — Source Data for Figure 4 [file MSB-14-e7985-s009.zip › Fig_4F_Part1_RAD53.tif]

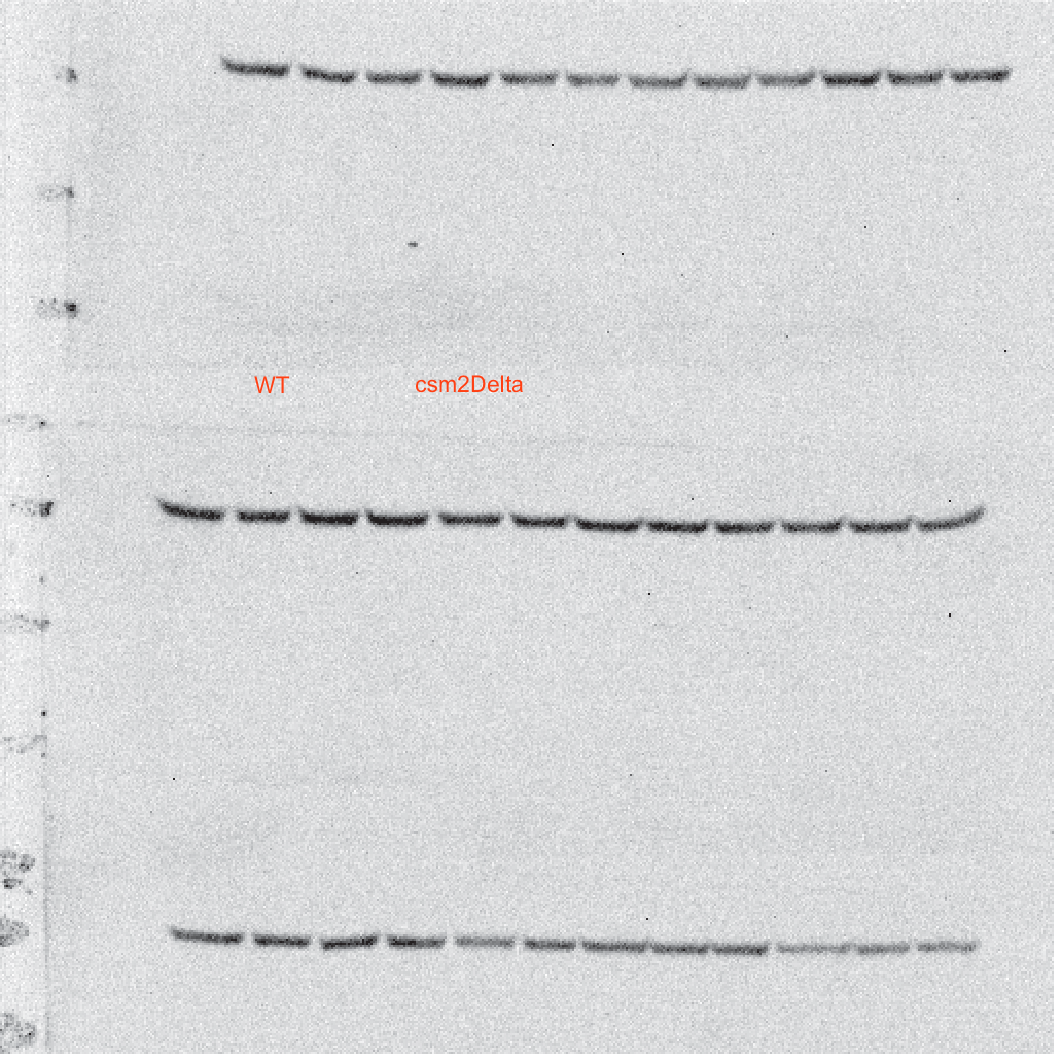

Supplement: Supplementary file 10 — Source Data for Figure 4 [file MSB-14-e7985-s009.zip › Fig_4F_Part2_PGK1.tif]

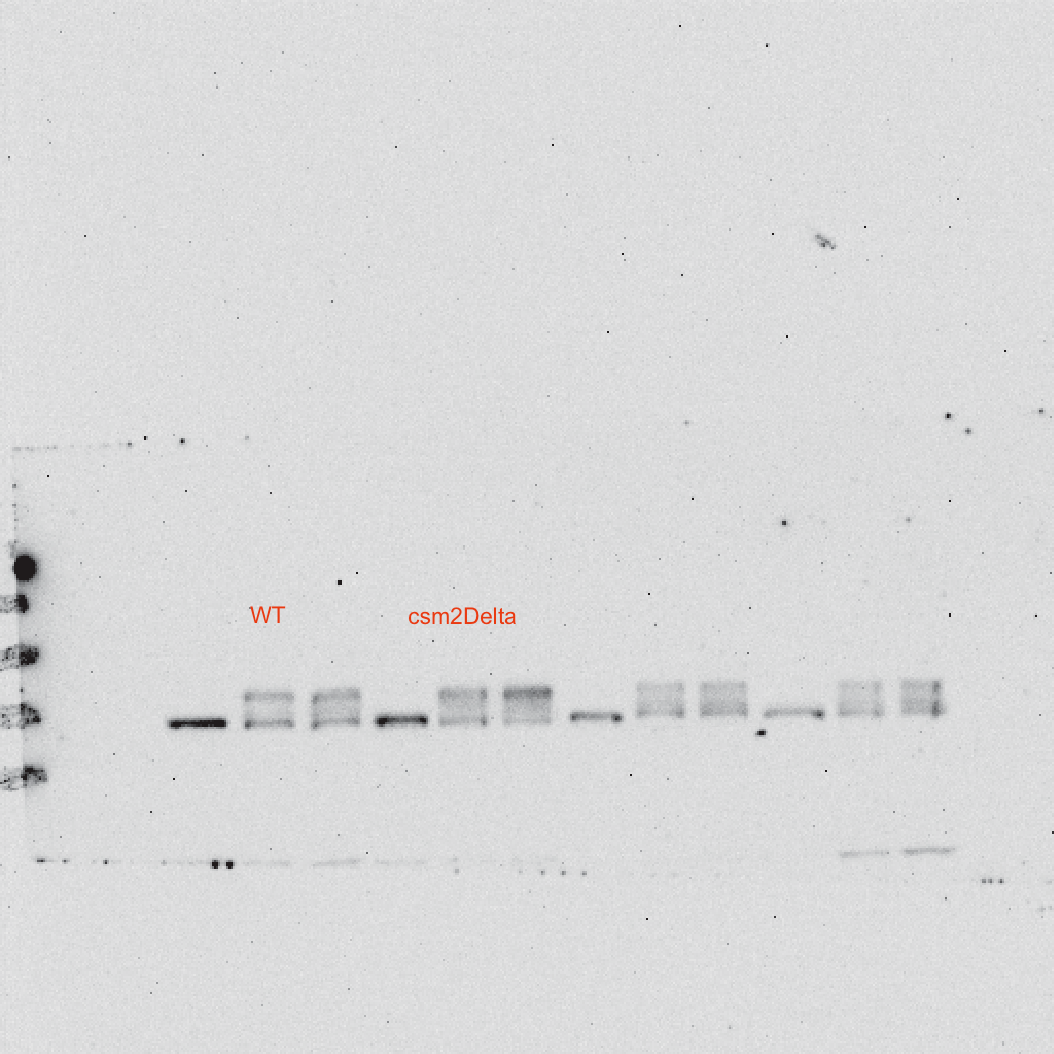

Supplement: Supplementary file 10 — Source Data for Figure 4 [file MSB-14-e7985-s009.zip › Fig_4F_Part2_RAD53.tif]
